# RNAIII is linked with the pentose phosphate pathway through the activation of RpiRc in *Staphylococcus aureus*

**DOI:** 10.1128/msphere.00348-23

**Published:** 2024-04-09

**Authors:** Marc Hallier, Julie Bronsard, Stéphane Dréano, Mohamed Sassi, Vincent Cattoir, Brice Felden, Yoann Augagneur

**Affiliations:** 1 QCPS (Quality Control in Protein Synthesis), IGDR UMR CNRS 6290, Université de Rennes 1, Rennes, France; 2 BRM (Bacterial Regulatory RNAs and Medicine), UMR_S 1230, Université de Rennes 1, Rennes, France; 3 Molecular Bases of Tumorigenesis: VHL Disease Team, CNRS UMR 6290 IGDR, BIOSIT, Université de Rennes 1, Rennes, France; University of Nebraska Medical Center College of Medicine, Omaha, Nebraska, USA

**Keywords:** *Staphylococcus aureus*, RNAIII, MAPS, pentose phosphate pathway, metabolism, PPP

## Abstract

*Staphylococcus aureus* RNAIII is a dual-function regulatory RNA that controls the expression of multiple virulence genes and especially the transition from adhesion to the production of exotoxins. However, its contribution to *S. aureus* central metabolism remains unclear. Using MS2-affinity purification coupled with RNA sequencing, we uncovered more than 50 novel RNAIII-mRNA interactions. Among them, we demonstrate that RNAIII is a major activator of the *rpiRc* gene, encoding a regulator of the pentose phosphate pathway (PPP). RNAIII binds the 5′ UTR of *rpiRc* mRNA to favor ribosome loading, leading to an increased expression of RpiRc and, subsequently, of two PPP enzymes. Finally, we show that RNAIII and RpiRc are involved in *S. aureus* fitness in media supplemented with various carbohydrate sources related to PPP and glycolysis. Collectively, our data depict an unprecedented phenotype associated with the RNAIII regulon, especially the direct implication of RNAIII in central metabolic activity modulation. These findings show that the contribution of RNAIII in *Staphylococcus aureus* adaptation goes far beyond what was initially reported.

**IMPORTANCE:**

*Staphylococcus aureus* is a major human pathogen involved in acute and chronic infections. Highly recalcitrant to antibiotic treatment, persistent infections are mostly associated with the loss of RNAIII expression, a master RNA regulator responsible for the switch from colonization to infection. Here, we used the MS2 affinity purification coupled with RNA sequencing approach to identify novel mRNA targets of RNAIII and uncover novel functions. We demonstrate that RNAIII is an activator of the expression of genes involved in the pentose phosphate pathway and is implicated in the adjustment of bacterial fitness as a function of carbohydrate sources. Taken together, our results demonstrate an unprecedented role of RNAIII that goes beyond the knowledge gained so far and contributes to a better understanding of the role of RNAIII in bacterial adaptation expression and the coordination of a complex regulatory network.

## INTRODUCTION

*Staphylococcus aureus* is a major community- and hospital-acquired pathogen. It causes a wide spectrum of infections, ranging from benign skin and soft tissue infections to fatal invasive diseases (1, 2). To succeed, *S. aureus* has to adapt to multiple environmental changes. To that end, it has a complex regulatory network that allows a fine adjustment of gene expression. For instance, a functional *agr* system, whose expression is based on cell density (3), is critical for full expression of virulence factors and to subvert the host innate immunity (4). A particularity of this quorum-sensing (QS) system is that the transcription factor AgrA, encoded by the *agr* system, activates both the *agr* P2 operon (*agrBDCA*), allowing autoinduction of the system, and the adjacent P3 promoter encoding RNAIII (5).

RNAIII is one of the main intracellular effectors of the *agr* QS system. It is a 514 nt-long regulatory RNA that possesses an intricate folding composed of 14 stem-loops (H1–H14) and three long-distance helices (6). It also encodes the δ-hemolysin peptide, which displays hemolytic and antimicrobial activities. By antisense pairings, RNAIII regulates the expression of many targets at multiple levels, including transcription, translation, and mRNA stability (7). Through various structural domains, RNAIII acts as both an activator and a repressor of dedicated mRNA targets to induce the expression of extracellular virulence factors while repressing the expression of cell wall-associated proteins (8). More than a dozen targets were identified, and their roles and mechanisms of action were extensively studied. RNAIII regulates the expression of many surface adhesion proteins (Eap and Sa1000) and host immune evasion proteins (Spa, Sbi, and EcB) (9–13). It affects the cell wall integrity by repressing LytM (14), a hydrolase involved in peptidoglycan turnover, and LtaS, an enzyme required for the synthesis of lipoteichoic acid (LTA), one of the major components of the cell wall (15). RNAIII positively regulates the expression of *hla*, encoding α-hemolysin (16), and indirectly activates toxin production by inhibiting the translation of *rot* mRNA, encoding the repressor of toxin Rot (10, 17). The regulation of RNAIII expression favors a switch between early expression of surface proteins that facilitate colonization and tissue invasion and late production of exotoxins that contribute to the establishment of the infection. Also, its downregulation suggests that it may be an important factor in establishing persistent cells (18–20).

Although RNAIII is most often studied as a regulator, some transcription factors (TF) involved in metabolic pathways were shown to regulate its expression. For instance, the inactivation of *rpiRc*, involved in the pentose phosphate pathway (PPP), leads to an increased expression of RNAIII during the exponential phase of growth (21). Similarly, inactivation of *codY,* encoding a repressor of factors required for adaptation to poor nutritional conditions (22), derepresses the synthesis of the *agr* locus and thus indirectly causes overexpression of RNAIII during exponential growth in rich medium (23). The two-component regulatory system SrrAB, which controls the downregulation of TCA cycle enzymes under anaerobic conditions, represses the expression of RNAIII by directly binding onto the *agr* promoter (24, 25). Inactivation of the catabolite control protein A (CcpA), an important global regulator of carbon catabolite repression (26, 27), results in a lower level of RNAIII only in the early-stationary phase of growth (28). It thus appears that there is a complex network between the metabolic status of *S. aureus*, their regulators, and the expression of RNAIII. Conversely, there are no known direct targets of RNAIII, allowing us to connect RNAIII with a functional role in the regulation of these metabolic pathways in *S. aureus*.

In this study, we used MS2 affinity purification coupled with RNA sequencing (MAPS) to expand the RNAIII targetome. We identified several novel targets and thoroughly studied one of them, encoding the transcription factor RpiRc involved in PPP. We showed that RNAIII is required for full expression of the RpiRc protein. Taken together, our study delineates a novel role for RNAIII in controlling metabolic pathways.

## RESULTS

### Identification of novel RNAIII targets by MAPS

To identify novel RNAIII targets, we performed MS2 affinity purification coupled with RNA sequencing (29) in wild-type *S. aureus* HG003 strain. MS2-tagged RNAIII or MS2 alone were expressed transiently in the early-stationary growth phase by using an anhydrotetracycline-inducible promoter. Two MS2 RNA aptamers were fused to the 5′ end of RNAIII, allowing RNAIII purification on MS2-maltose-binding protein immobilized on amylose beads. A 5-min induction led to around 10-fold overexpression of MS2-tagged RNAIII compared to endogenous RNAIII (Fig. S1A). Crude extracts from HG003 strains expressing MS2-tagged RNAIII or MS2 alone were passed through MS2-Maltose resin. Following several washes, tagged sRNA and associated RNAs were eluted and purified. Most of the MS2-tagged RNAIII was purified through this affinity chromatography, indicating efficient binding to resin and recovery of RNAIII-target complexes (Fig. S1B). Eluted RNAs were subjected to RNAseq, and the enrichment of mRNAs upon MS2-RNAIII induction versus MS2 alone was determined. DESeq analysis allowed the identification of 53 candidates, among which several known targets of RNAIII were recovered (Table S1). These include mRNAs coding for hemolysin α and transcriptional factors Rot and MgrA with around 8-, 10-, and 35-fold enrichment, respectively. Then, we focused our analyses on one of the 10 most relevant RNAIII targets (Table 1), which is *rpiRc* with a 113-fold increase.

**TABLE 1:** List of RNAs significantly co-purified with MS2-RNAIII^*a*^

Gene ID	Gene name	Product	RNA fold change	*P*-value
SAOUHSC_01024	*graF*	Glycopeptide resistance-associated gene F	443.75	1.0139E-07
SAOUHSC_02589	*rpiRc*	Phosphosugar-binding transcriptional regulator, RpiRc	112.98	0.00011283
SAOUHSC_02663	_	Uncharacterized protein	59.05	0.00011283
SAOUHSC_01174	_	Uncharacterized protein	57.05	6.5725E-05
SAOUHSC_01462	*gpsB*	Cell division protein	39.78	2.1157E-05
SAOUHSC_01730	*csbD*	Controlled by SigmaB (uncharacterized protein)	37.27	2.8383E-05
SAOUHSC_02850	*cidB*	Holin-like protein CidB	37.26	1.0904E-05
SAOUHSC_00694	*mgrA*	HTH-type transcriptional regulator MgrA	36.9	0.00014279
SAOUHSC_00617	_	Uncharacterized protein	36.44	6.6204E-10
SAOUHSC_02656	_	Uncharacterized protein	32.57	9.6209E-07

^*a*^ RNA fold change and *P*-values were calculated from three independent experiments. The cut-off of enrichment and the *P*-value were fixed at 30 and <0.0002, respectively.

**Fig 1:**
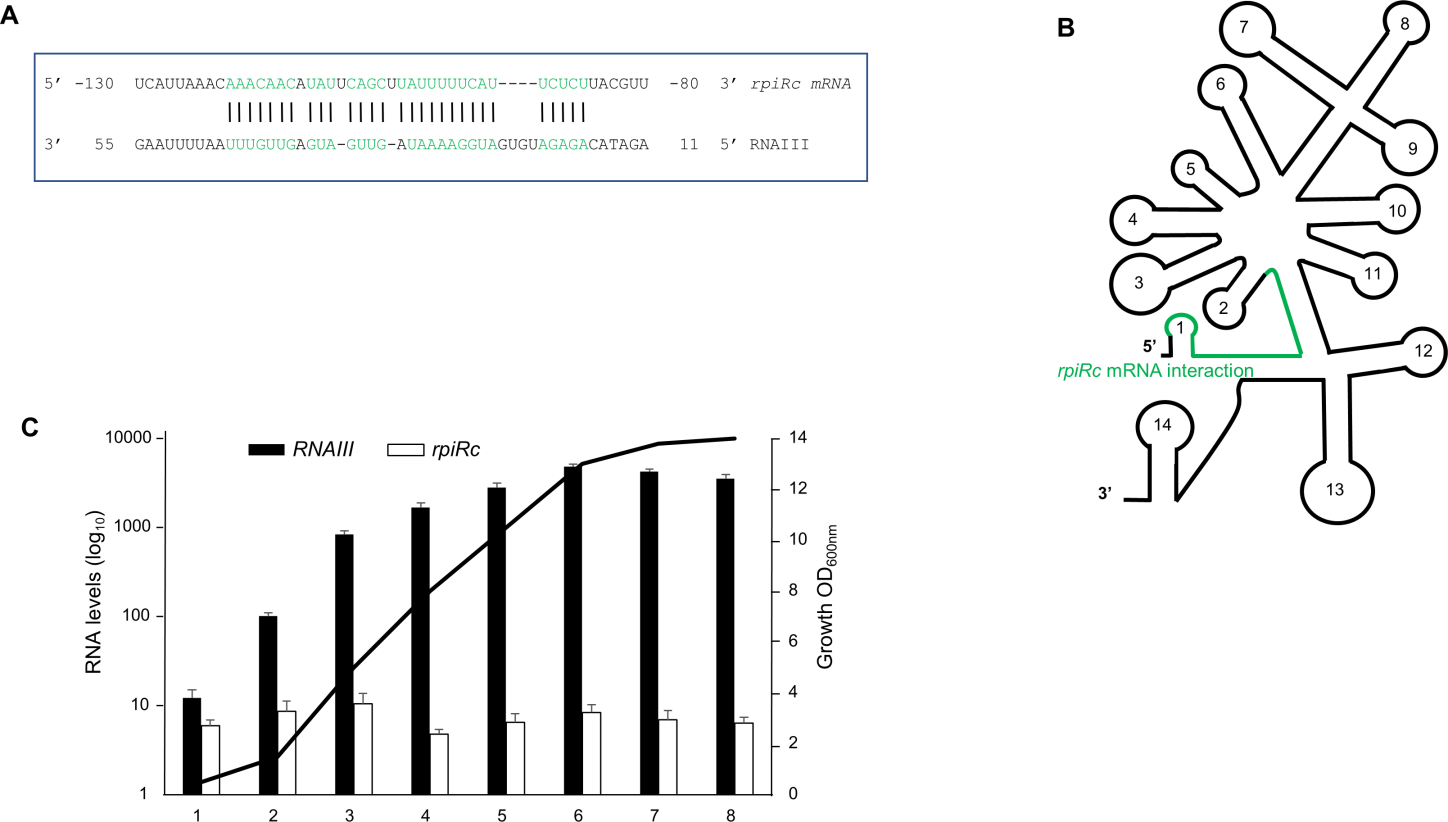
Putative interactions between RNAIII and *rpiRc* mRNA, and RNA expression profiles. (**A**) Base-pairing predictions between RNAIII and *rpiRc* mRNA using IntaRNA software. The predicted Δ*G* of the interaction is −17.78 kcal/mol. (**B**) Location of RNAIII region involved in interaction with *rpiRc* mRNA. RNAIII region potentially interacting with *rpiRc* is shown in green. The hairpin structures of RNAIII are numbered from 1 to 14. (**C**) Expression profiles of RNAIII and *rpiRc* mRNA during bacterial growth in brain heart infusion medium. The relative expressions were quantified by quantitative real-time PCR in the HG003 strain. The data were normalized to the level of *gyrB* mRNA. Shown are the mean and standard deviation for three independent experiments. Due to the high expression of RNAIII compared to *rpiRc*, the RNA levels are represented in a logarithmic scale. The cell density was monitored at 600 nm, starting from an OD_600nm_ of 0.1. The growth curve shown is one representative experiment among the three.

The intaRNA program from Freiburg RNA tools was used to predict interactions between RNAIII and *rpiRc* mRNA. A putative base-pair annealing was found with robust minimal energy (−17.78 kcal/mol) (Fig. 1A). The 5′ region of RNAIII was predicted to bind the 5′ UTR of *rpiRc* mRNA (Fig. 1B). Base pairing complementarities were predicted between nucleotides −86 and −118 at the 5′ end of *rpiRc* with nucleotides + 12 to +46 of RNAIII (Fig. 1A and B). This 5′ end domain of RNAIII is known to be directly involved in the regulation of the expression of *mgrA* and *hla* genes (16, 17, 30). To anticipate whether RNAIII could be a potent regulator of *rpiRc*, we monitored their expression along with their growth. Overall, the RNAIII level was from 2- to 600-fold higher than that of *rpiRc* mRNA (Fig. 1C). *rpiRc* mRNA was constitutively expressed during bacterial growth, whereas RNAIII expression pattern was typical with an expression linked with QS. Altogether, the prediction of the interaction domains and the RNA expression profiles suggest that RNAIII is likely a regulator of *rpiRc*.

### RNAIII upregulates *rpiRc* expression

**Fig 2:**
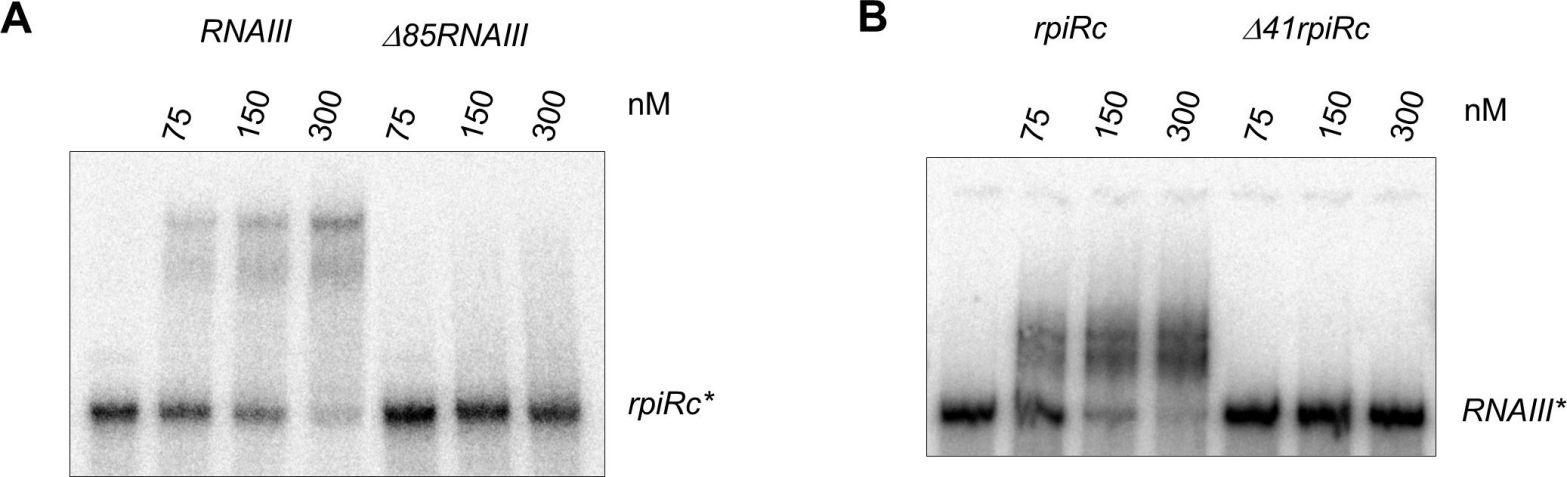
Gel retardation assay to monitor RNAIII binding to *rpiRC* mRNA. (**A**) The 5′ end-labeled *rpiRc* mRNA (nucleotides −161 to +45) was incubated with increasing concentrations (nM) of RNAIII or RNAIII devoid of its first 85 nucleotides. The apparent Kd value (200 nM) was determined as the concentration of RNAIII allowing 50% of *rpiRC* mRNA binding. (**B**) The 5′ end-labeled RNAIII was incubated with increasing concentrations (nM) of *rpiRc* mRNA (nucleotides −161 to +45) or *rpiRc* mRNA lacking the sequence predicted to interact with RNAIII (*Δ41rpiRc*).

The *rpiRc* mRNA encodes a TF involved in the regulation of the PPP and RNAIII expression (21). Our analysis of *rpiRc* mRNA 5′ end by circular RT-PCR and Sanger sequencing revealed that *rpiRc* mRNA contains a long 5′ UTR of 324 nucleotides (Fig. 2). We first confirmed that RNAIII directly binds *rpiRc* mRNA using gel retardation assay (Fig. 2). RNAIII interacts with a *rpiRc* transcript containing 161 nucleotides of the 5′ UTR and 45 nucleotides of the coding region with a Kd of ≈ 200 nM (Fig. 2A and B). The interaction is specific since no gel retardation was observed with RNAIII deprived of its 5′ end, which contains the predicted domain of interaction with *rpiRc* mRNA (Δ85RNAIII) (Fig. 2A), and with a *rpiRc* transcript lacking its predicted pairing region with RNAIII (*Δ41rpiRc*) (Fig. 2B).

**Fig 3:**
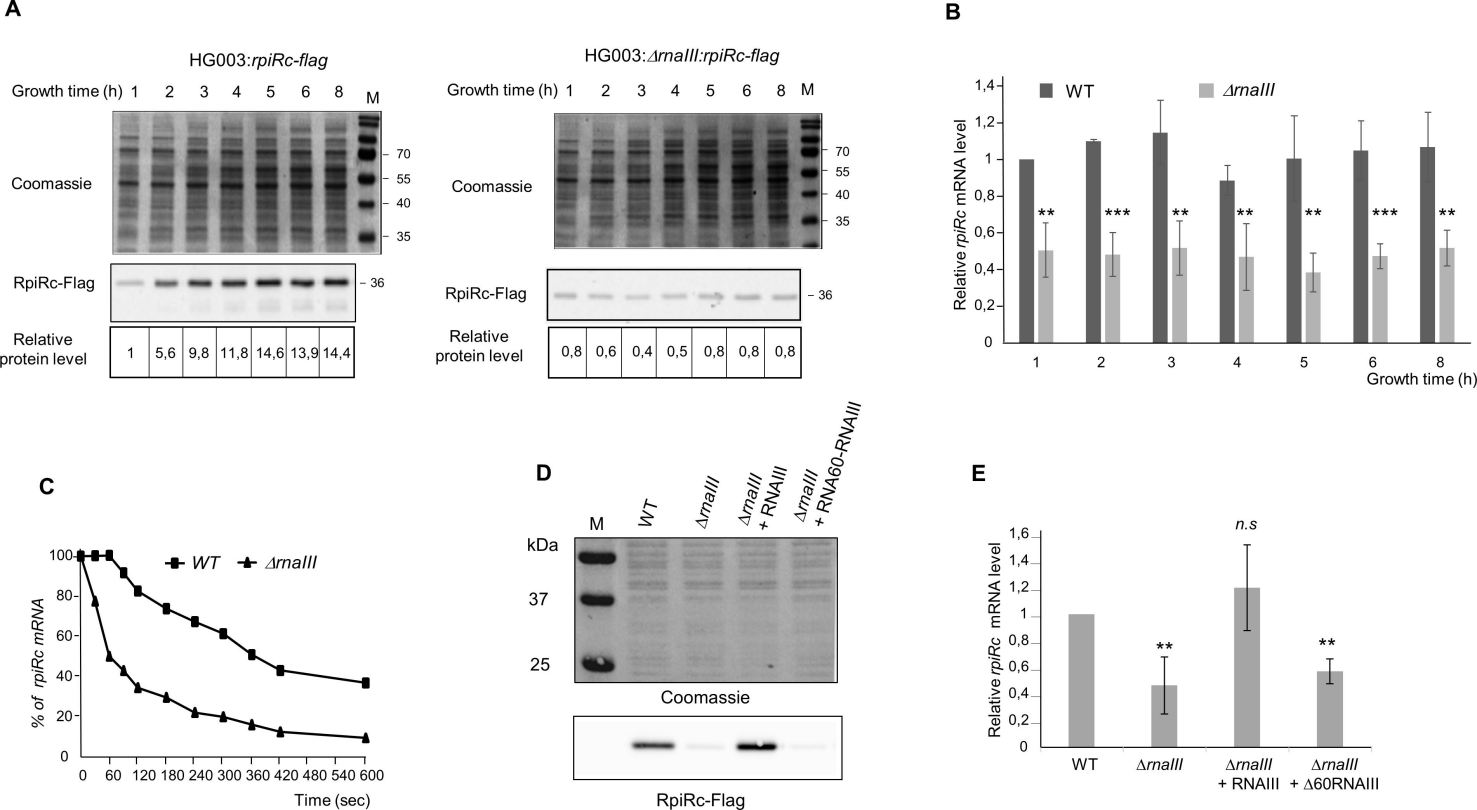
RNAIII is required for full expression of *rpiRc*. (**A**) Expression profile of endogenous FLAG-tagged RpiRc proteins in HG003:*rpiRc-flag* and HG003:Δ*rnaIII:rpiRc-flag* strains. FLAG-tagged RpiRc proteins were detected by immunoblotting with anti-FLAG antibodies. The relative expression of RpiRc-FLAG was normalized to 1 in HG003:*rpiRc-flag* strain HG003 after 1 h of growth. (**B**) Expression profile of *rpiRc* mRNA in HG003 and HG003:Δ*rnaIII* strains. The relative expressions of *rpiRc* in the HG003 and HG003:Δ*rnaIII* strains were quantified by quantitative real-time PCR (RT-qPCR). The data were normalized to the level of *gyrB* mRNA. The bars represent the fold change of the expression of *rpiRc* relative to the expression of *rpiRc* in the strain HG003 at 1 h of growth. Shown are the mean and standard deviation (SD) for three independent experiments. (**C**) Determination of *rpiRc* mRNA half-life by RT-qPCR in the presence of rifampicin (400 µg/mL) in the HG003 and HG003:Δ*rnaIII* strains. The half-life was given as the time when 50% of *rpiRc* mRNA was degraded. The relative transcript levels were normalized to *tmRNA*. Shown are the means for three independent experiments. (**D**) Western blot analysis demonstrating that plasmid-based complementation of RNAIII expressed under its endogenous promoter (+RNAIII) totally restores the expression of FLAG-tagged RpiRc protein in the early-stationary phase of growth (6 h). RNAIII lacking its *rpiRc* interacting domain (+Δ60RNAIII) is not able to restore the expression level of endogenous FLAG-tagged RpiRc proteins in the HG003:Δ*rnaIII* strain. Coomassie blue-stained SDS-PAGE gel is included to demonstrate the equivalent loading of total proteins. (**E**) RNAIII modulates *rpiRc* mRNA expression. The *rpiRc* mRNA was quantified by RT-qPCR. The data were normalized to the level of *gyrB* mRNA expression from total extracts prepared from culture to the early-stationary phase (6 h) of HG003 (WT), the Δ*rnaIII* strain, and the Δ*rnaIII* strains complemented with a plasmid expressing either RNAIII or Δ60RNAIII under the endogenous RNAIII promoters (pISC3-*rnaIII* and pISC3-*Δ60rnaIII*). The bars represent the fold change of the expression of *rpiRc* relative to its expression in the wild-type HG003 strain. Shown are the mean and SD for three independent experiments. Student’s *t*-test was applied to calculate if the differences are statistically significant (**P < 0.01), highly significant (***P < 0.001), or not significant (n.s., *P* > 0.05).

To find out whether RNAIII could modulate the expression level of *rpiRc*, we introduced a FLAG epitope at the 3′ end of the *rpiRc* gene into the chromosome of HG003 and HG003:Δ*rnaIII* strains. Immunoblotting, using an anti-flag antibody, revealed that the level of RpiRc protein was drastically reduced throughout bacterial growth in the HG003Δ*rnaIII::rpiRc-flag* strain (Fig. 3A, right panel). In the HG003::*rpiRc-flag* strain, we observed a correlation between the expression levels of RpiRc protein and RNAIII during bacterial growth (Fig. 3A and 1C). The expression of the RpiRc protein increased considerably after the exponential phase of growth when the expression of RNAIII was the highest. This accumulation of RpiRc protein appeared mainly linked to a modulation of the translation of *rpiRc* mRNA since the level of *rpiRc* transcript remained constant during growth (Fig. 3B). However, in the absence of RNAIII, the *rpiRc* mRNA levels were reduced by around twofold, and the RpiRc protein continuously expressed at a very weak level (Fig. 3A and B). To decipher whether RNAIII plays a role in *rpiRc* mRNA stability, we measured its half-life. This showed that whereas the half-life of *rpiRc* mRNA was around 6 min in the parental strain, it dropped to only 1 min in HG003Δ*rnaIII* (Fig. 3C).

Additionally, RpiRc protein and *rpiRc* mRNA levels were completely restored in strain HG003*ΔrnaIII::rpiRc-flag,* in which a full-length RNAIII was expressed at a level slightly higher than that found in the WT strain (Fig. S3), but not when complemented with Δ60-RNAIII, an RNAIII deprived of its *rpiRc* interaction domain (Fig. 3D and E), confirming that the upregulation of the *rpiRc* gene expression involves a direct interaction of the 5′ end of RNAIII with the 5′ UTR of *rpiRc* mRNA. Altogether, these data show that RNAIII, and especially its first hairpin, is required to increase both the translation and the stability of *rpiRc* mRNA.

### RNAIII activates *rpiRc* translation and stabilizes *rpiRc* mRNA

**Fig 4:**
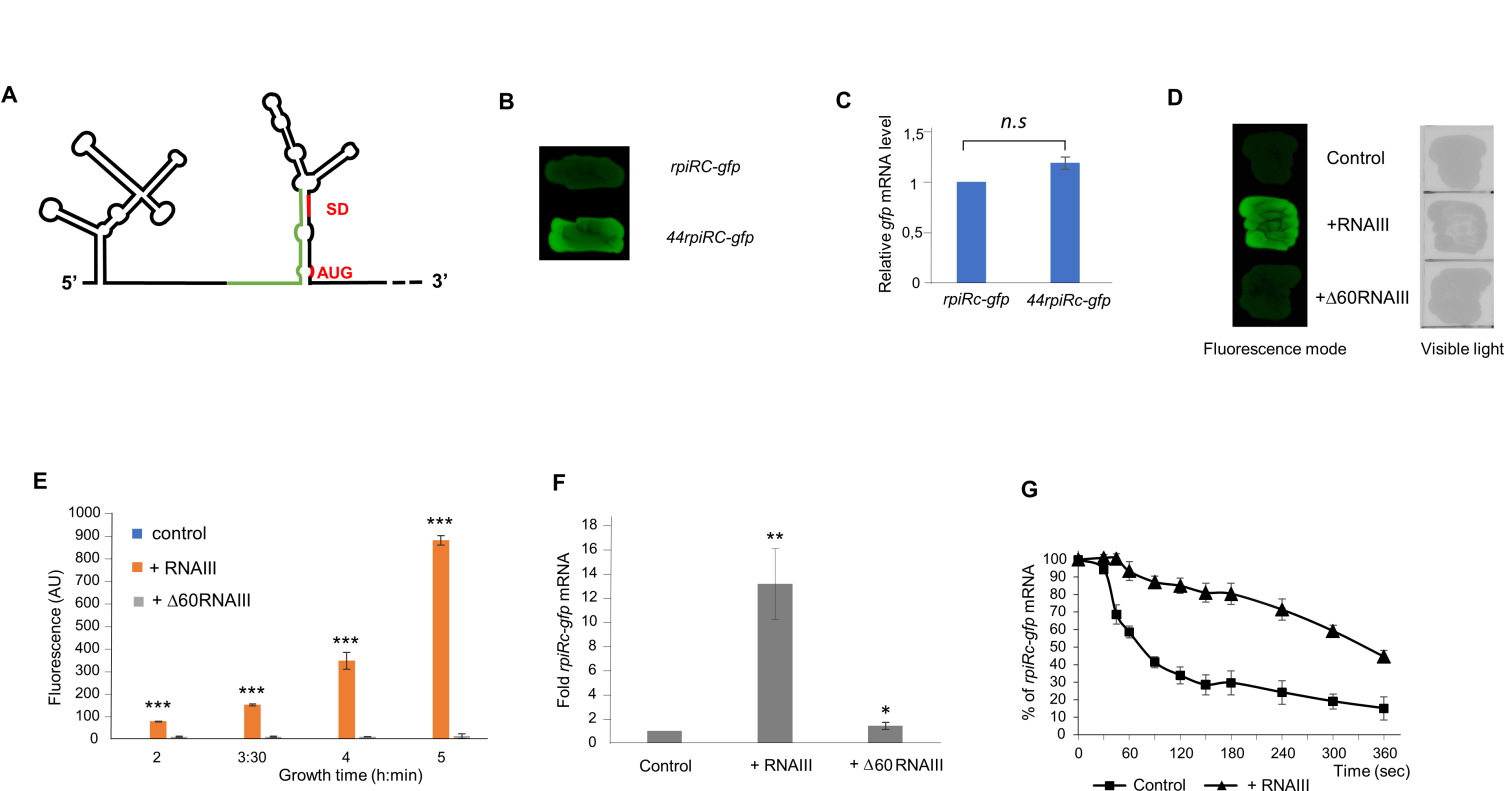
RNAIII improves the translation of *rpiRc* mRNA. (**A**) Secondary structure model of the 5′ region of *rpiRc* mRNA by using the mfold program. The Shine-Dalgarno sequence and AUG start codon are indicated in red. The *rpiRc* region interacting with RNAIII is green. (**B**) The highly structured 5′ UTR of *rpiRc* impairs the translation of *rpiRc-gfp* mRNA. Fluorescence of HG003:*ΔrnaIII* strains expressing either *rpiRc-gfp* (containing the full length 5′ UTR of *rpiRc* mRNA) or *44rpiRc-gfp* (containing a shortened 5′ UTR of 44 nucleotides) under the control of P*tufA* promoter were monitored on brain heart infusion (BHI) agar plates supplemented with erythromycin (2.5 µg/mL). (**C**) RT-qPCR analysis of *rpiRc-gfp* and *44rpiRc-gfp* mRNA expression levels using specific *gfp* primers. The bars represent the fold change of the expression of *gfp* in the HG003:ΔRNAIII strain at 6 h of growth. Shown are the mean and standard deviation (SD) for three independent experiments. (**D and E**) Fluorescence stimulation in HG003:*ΔrnaIII* cells expressing the *rpiRc-gfp* fusion when co-expressed with RNAIII. HG003:*ΔrnaIII* strains carrying the pCN33*-rpiRc-gfp* fusion plasmid under the control of P*tufA* promoter were co-transformed with pISC3 (control), pISC3-P*amiA-rnaIII*, or pISC3-P*amiA-Δ60rnaIII* and were grown on BHI agar plates (**D**) or in liquid BHI medium (**E**) supplemented with chloramphenicol (10 µg/mL) and erythromycin (2.5 µg/mL). Fluorescence levels were not stimulated when RNAIII was mutated in the domain interacting with *rpiRc* mRNA (Δ60RNAIII). Growth (OD_600nm_) and fluorescence were measured as described in Fig. 4. The control of bacterial growth on BHI agar plates was obtained using visible light. (**F**) Activation of *rpiRc* expression measured by quantitative real-time PCR (RT-qPCR) and using *rpiRc-gfp* fusions. Relative expression was monitored using specific *gfp* primers in HG003:*ΔrnaIII* cells expressing pCN33*-*P*tufA-rpiRc-gfp* and pISC3 (control), pISC3-P*amiA-rnaIII,* or pISC3-P*amiA-Δ60rnaIII* plasmids. Relative expressions of *rpiRc-gfp* mRNA under the P*tufA* promoter are expressed as fold change compared to the control cell. Student’s *t*-test was applied to calculate whether the differences are statistically significant (*P < 0.05), highly significant (**P < 0.01 and ***P < 0.001), or not significant (n.s., *P* > 0.05). (**G**) Half-life determination of *rpiRc-gfp* mRNA measured by RT-qPCR in the presence of rifampicin (400 µg/mL) in the strains described in panel **F**. The half-life was given as the time when 50% of *rpiRc-gfp* mRNA was degraded. The relative transcript levels were normalized to *tmRNA*. Shown are the mean and SD for three independent experiments.

Prediction of the secondary structure of the 5′ region of *rpiRc* mRNA using the mfold program revealed that the 5′ UTR and the translational initiation site were highly structured (Fig. 4A). The Shine Dalgarno (SD) and the initiation codon (AUG) are in a strong hairpin structure, which could render the translation initiation site inaccessible. To determine whether the regulation of *rpiRc* gene expression by RNAIII is mainly at the translational level and occurs at this 5′ UTR, translational fusions to GFP were cloned into the low-copy pCN33-*gfp* vector under a constitutive P*tufA* promoter in HG003*ΔrnaIII* strain. While the presence of a full-length 5′ UTR sequence led to a moderate level of fluorescence, the use of a shortened 5′ UTR of 44 nucleotides containing the SD sequence of *rpiRc* mRNA dramatically increased the production of GFP protein (Fig. 4B). Study of *gfp* mRNA level showed similar transcript levels, suggesting that the highly structured 5′ UTR sequence appears to prevent the translation of the *rpiRc* mRNA (Fig. 4C). A similar increase in GFP was observed, both in Petri dishes and in liquid cultures, when RNAIII was overexpressed in cells expressing the entire 5′ UTR of *rpiRc* fused to *gfp* (Fig. 4D and E). This increase in GFP expression is related to the interaction of RNAIII with the 5′ UTR of *rpiRc* since overexpression of Δ60-RNAIII, which is deleted for the *rpiRc* interaction domain, had no effect on target production (Fig. 4D and E). Therefore, these results suggested that RNAIII favors *rpiRc* mRNA translation initiation and could modify the structure of the 5′ UTR of *rpiRc*. We used toeprint to determine whether full-length RNAIII could facilitate the formation of a ternary initiation complex formed between the *S. aureus* ribosome, initiator tRNA^Met^, and *rpiRc-gfp* mRNA (Fig. S4). Formation of the ternary complex blocked the elongation of a cDNA primer by reverse transcriptase and produced a toeprint around 15 nucleotides downstream from the initiation codon. The binding of RNAIII to *rpiRc* mRNA strongly increased the toeprint signal and led to structural modifications within the whole 5′ UTR (Fig. S4, lane 3). Conversely, the toeprint did not vary in the presence of an RNAIII deprived of its interaction domain with *rpiRc* (*Δ85RNAIII*). Therefore, our results show that RNAIII favors *rpiRc* mRNA translation initiation by binding the 5′ UTR upstream of the SD to modify the secondary structure and increase ribosome loading.

To assess whether RNAIII could also affect the *rpiRc* mRNA level, the steady state and stability of *rpiRc-gfp* mRNA were monitored (Fig. 4F and G). The amount of *rpiRc-gfp* mRNA produced from the constitutive P*tufA* promoter was increased when RNAIII was overexpressed (Fig. 4F). Conversely, the overexpression of Δ60RNAIII did not alter the *rpiRc-gfp* mRNA level (Fig. 4F). The effect of RNAIII on *rpiRc* gene expression appears to be mainly at the translational level since the RpiRc-GFP protein level was increased by a factor of about 250 when RNAIII was overexpressed (Fig. 4E), whereas *rpiRc-gfp* mRNA production only increased by around 12 (Fig. 4F). Half-life determination of *rpiRc-gfp* mRNA in the presence or absence of RNAIII revealed that RNAIII increased *rpiRc-gfp* mRNA level by changing mRNA stability (Fig. 4G). In accordance with the RNAIII-dependent stability of endogenous *rpiRc* mRNA (Fig. 3C), the half-life of *rpiRc-gfp* mRNA was around 1 min in HG003Δ*rnaIII* strain but increased to 5 min 30 s in the presence of RNAIII. Altogether, our data showed that RNAIII binds to the 5′ UTR of *rpiRc* mRNA to favor translation and, to some extent, stabilizes the *rpiRc* mRNA.

### RNAIII activates the expression of genes involved in PPP

**Fig 5:**
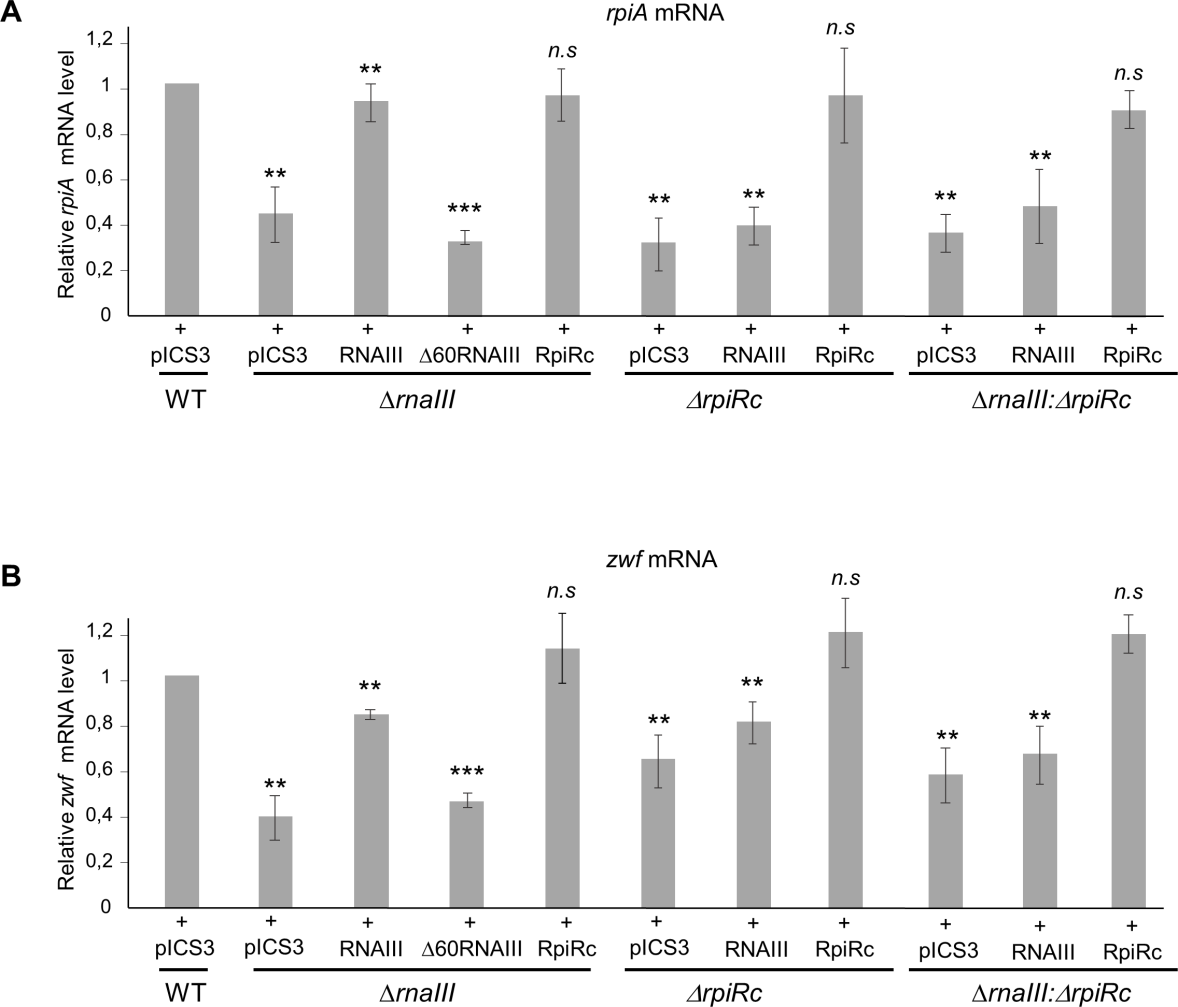
RNAIII modulates *rpiA* and *zwf* mRNA expressions. The *rpiA* (**A**) and *zwf* (**B**) mRNAs were quantified by RT-qPCR from the culture in the exponential phase of growth (2 h) in tryptic soy broth. Transcript levels were monitored in HG003 (WT), Δ*rnaIII*, Δ*rpiRc,* and Δ*rnaIII:*Δ*rpiRc* strains complemented with pISC3 plasmids expressing either RNAIII, Δ60RNAIII, or RpiRc under the endogenous *rnaIII* or *rpiRc* promoters. The data were normalized to the level of *gyrB* mRNA expression. The bars represent the fold change of the expression of *rpiA* or *zwf* relative to their expressions in the wild-type HG003 strain. Shown are the mean and standard deviation for three independent experiments. Student’s *t*-test was applied to calculate if the differences are statistically significant (**P < 0.01 and ***P < 0.001).

RpiRc has a positive regulatory function in PPP. Indeed, the PPP genes *rpiA* (ribose-5-phosphate isomerase A) and *zwf* (glucose 6-phosphate dehydrogenase) are positively regulated by rpiRc (21). To determine whether the inactivation of RNAIII could deregulate the PPP by modifying the expression of *rpiRc* and therefore the *rpiRc* regulon, the transcriptional levels of *rpiA* and *zwf* were assessed by quantitative real-time PCR (RT-qPCR). RNAIII inactivation decreased by around twofold the *rpiA* and *zwf* transcript levels during the exponential phase of growth (Fig. 5A and B). Complementation of the HG003*:ΔrnaIII* with RNAIII but not with a Δ60RNAIII mutant restored the *rpiA* and *zwf* mRNA levels, confirming that the expression changes were due to the inactivation of *rnaIII* gene *(*Fig. 5A and B*).* Also, the *rpiA* and *zwf* mRNA levels were restored in the HG003:*ΔrnaIII* strain when RpiRc was overexpressed from a pISC3 plasmid, showing the implication of RpiRc. To strengthen our investigations, we monitored *rpiA* and *zwf* transcript levels in HG003*:ΔrpiRc* and HG003*:ΔrpiRc:ΔrnaIII*. For both, the target mRNA levels decreased. Noteworthy, the single and the double mutants were complemented by RpiRc, but the double mutant was not complemented by RNAIII, confirming the direct role of RpiRc in PPP and the indirect role of RNAIII. Overall, our data indicate that RNAIII is needed to ensure temporal control of *rpiRc* expression and fine-tune PPP.

### Global impact of RNAIII, RpiRc, and carbohydrates on *S. aureus* fitness

**Fig 6:**
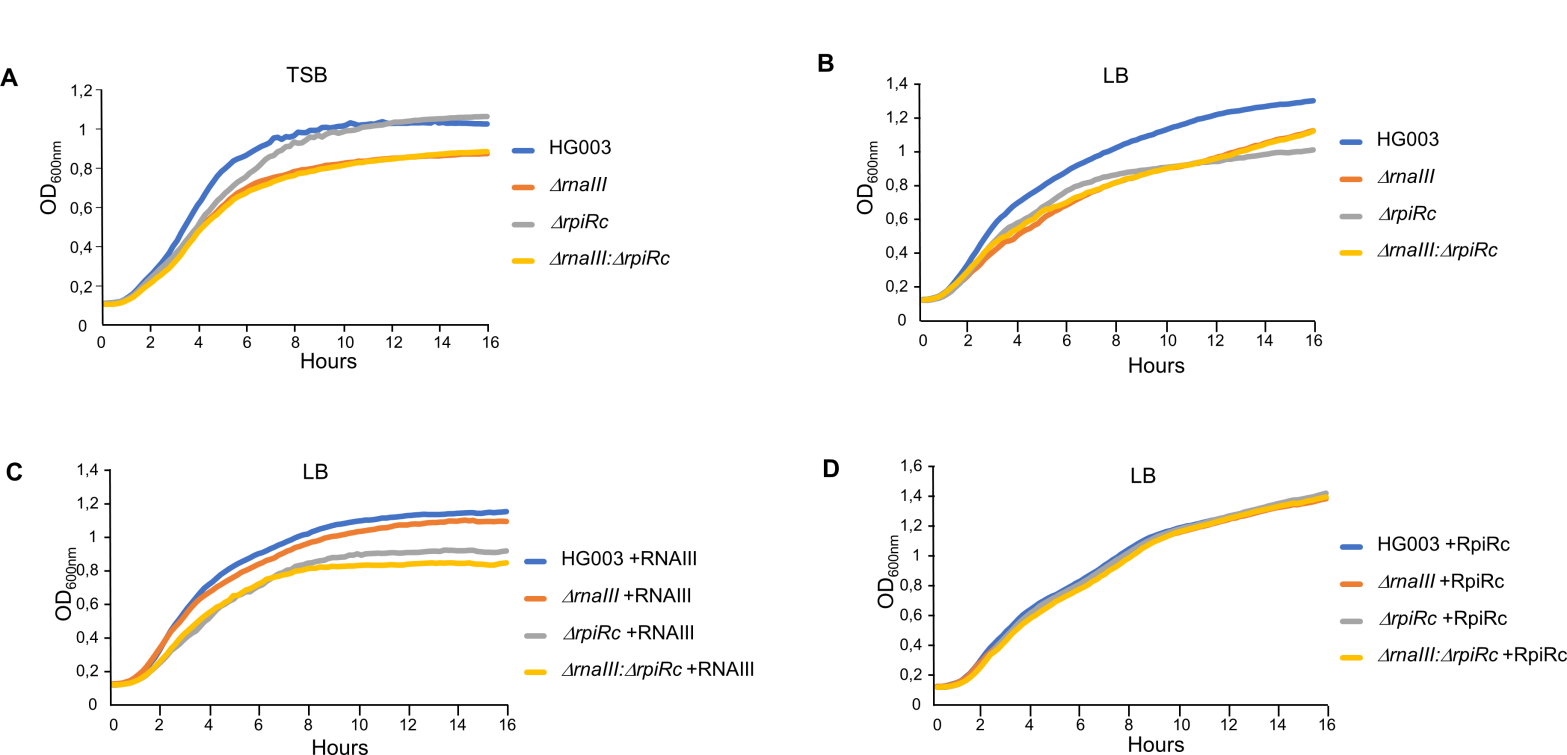
Overexpression of RpiRc restores the growth defect induced by the inactivation of *rpiRc* or *rnaIII*. (**A and B**) Growth of HG003, HG003:Δ*rnaIII* (Δ*rnaIII*), HG003:Δ*rpiRc* (Δ*rpiRc*), and HG003:Δ*rnaIII:*Δ*rpiRc* (Δ*rnaIII:*Δ*rpiRc*) strains in TSB (**A**) or in LB medium (**B**). (**C and D**) Growth of HG003 and its *rnaIII* and/or *rpiRc* mutants complemented with pICS3-*tufA-rnaIII* (+RNAIII) (**C**) or pICS3-*rpiRc* (+RpiRc) (**D**) in LB medium. Growth curves were obtained from overnight cultures that were diluted to an OD_600nm_ of 0.1. Optical density was measured every 10 min using Synergy 2 (BioTek). Each panel is a representative experiment of three.

**Fig 7:**
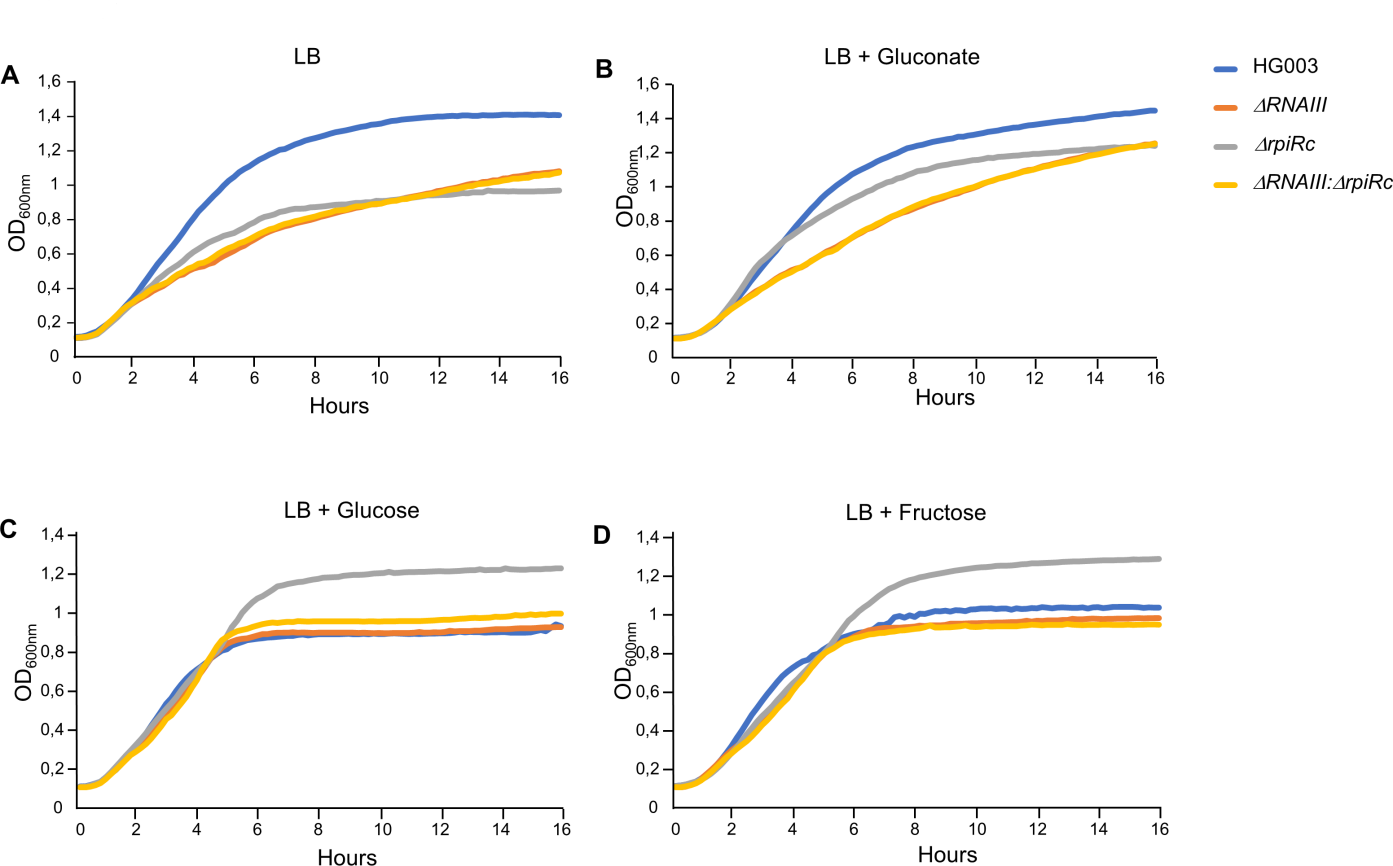
Effect of different carbon sources on the growth of HG003 mutants in LB medium. Growth of HG003, HG003:Δ*rnaIII* (Δ*rnaIII*), HG003:Δ*rpiRc* (Δ*rpiRc*), and HG003:Δ*rnaIII:*Δ*rpiRc* (Δ*rnaIII:*Δ*rpiRc*) strains in LB medium (**A**) supplemented with 0.5% of (**B**) gluconate, (**C**) glucose, or (**D**) fructose. Growth curves were obtained from overnight cultures that were diluted to an OD_600nm_ of 0.1. Optical density of growth was measured every 10 min using Synergy 2 (BioTek). Each panel is a representative experiment of three.

**Fig 8:**
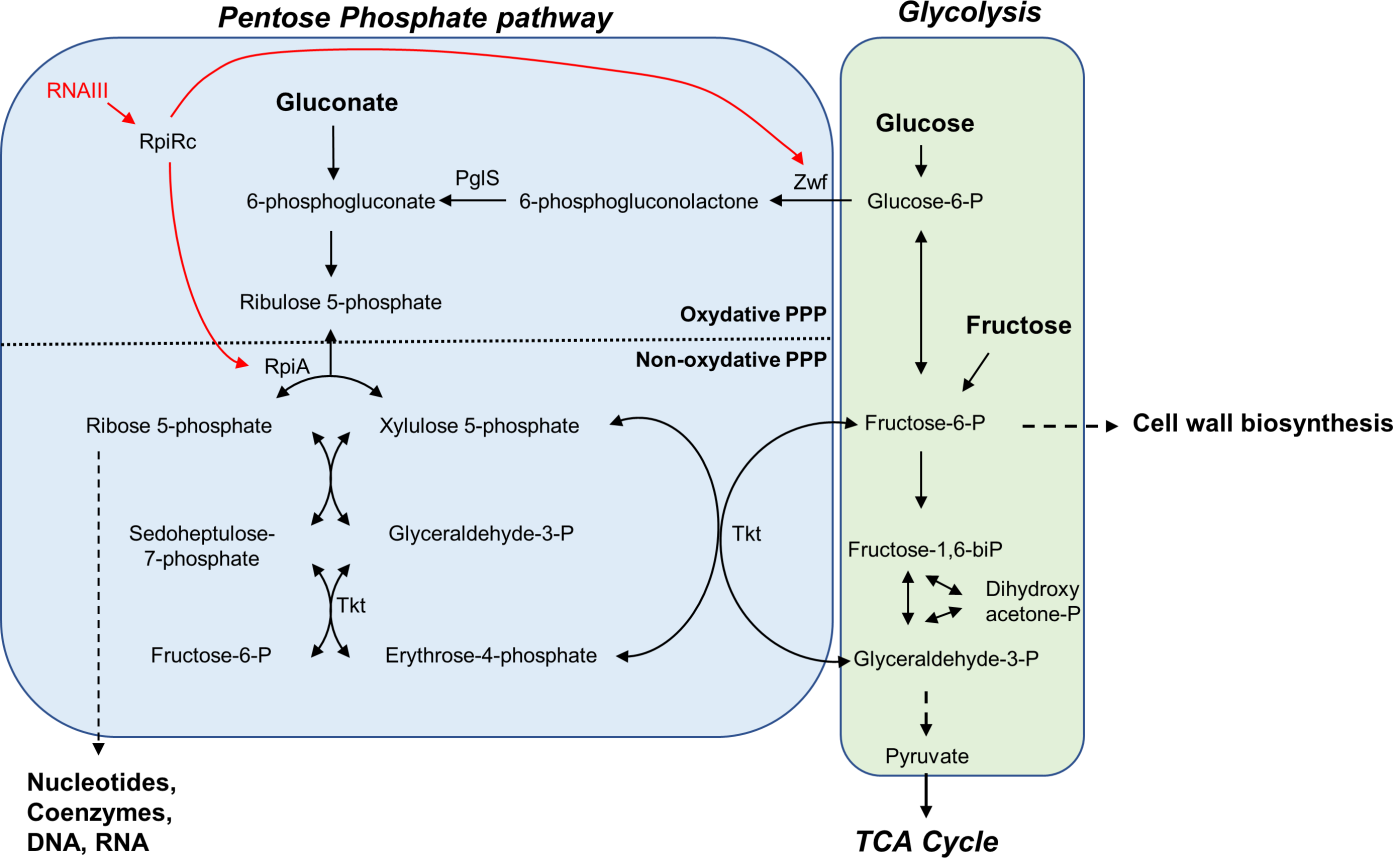
Schematic representation of the connections between the cell wall biosynthesis, the glycolytic and the pentose phosphate pathways, and the role of RpiRc and RNAIII to adjust metabolic fluxes. Key enzymes and metabolites are indicated. Carbon sources used in this study appear in bold.

Given the role of RpiRc in PPP (21) and our findings showing that RNAIII is a positive regulator of PPP, we investigated the contribution of RNAIII and/or RpiRc in bacterial growth in tryptic soy broth (TSB) and Lysogeny broth (LB) media (Fig. 6A and B). In TSB, the deletion of RNAIII led to a significant growth yield and rate defect. In turn, the HG003:*ΔrpiRc* strain exhibited an altered growth rate but the final biomass was similar to the parental strain. The double mutant behaved like the HG003:*ΔrnaIII* strain (Fig. 6A). In LB, the deletion of *rpiRc* had a stronger impact on both growth yield and rate defect, leading to a fitness similar to the *ΔrnaIII* or *ΔrnaIII:ΔrpiRc* mutants (Fig. 6B). Since growth in LB increased alterations in the mutant strains, we complemented all the strains with RNAIII expressed under the control of the *tufA* constitutive promoter (Fig. 6C) or RpiRc expressed under its endogenous promoter (Fig. 6D), which led to its overexpression (Fig. S5). Ectopic expression of RNAIII complemented HG003:*ΔrnaIII* growth defect but not HG003:*ΔrpiRc* and HG003:*ΔrnaIII:ΔrpiRc* mutants (Fig. 6C). Conversely, RpiRc complemented the growth defect of all three mutants, indicating an important role of RpiRc to adjust *S. aureus* fitness in LB medium (Fig. 6D). Therefore, we tested the effect of several carbohydrates on bacterial growth in LB (Fig. 7). First, when PPP was fueled with gluconate, allowing a bypass Zwf (Fig. 8), the HG003:*ΔrpiRc* mutant growth defect was complemented until the end of the exponential phase (Fig. 7B). Conversely, the growth of the two other strains (i.e., deprived of RNAIII) were not restored during the exponential phase, but the final biomasses formed were similar to the HG003:*ΔrpiRc* mutant and were closer to that of the parental strain (Fig. 7B).

When glucose or fructose were provided as the main carbon source, the fitness of the three mutants returned to close to that of the WT during the first 4 h of growth (Fig. 7C and D). As expected, the addition of glucose in LB drastically decreased the growth yield of the WT strain (Fig. 7A and C; Fig. S6) (31). Conversely, the growth yield of the HG003:*ΔrpiRc* mutant was significantly improved compared with the parental strain (Fig. 7C and D; Fig. S6). Whereas glucose improved the yield of the HG003*:ΔrpiRc* mutant, the two other mutants (i.e., in which *rnaIII* was deleted) show a growth close to the parental strain (Fig. 7C and D; Fig. S6). Also, in the presence of glucose, ectopic expression of RpiRc in the HG003*:ΔrpiRc* mutant restored parental growth (Fig. S7B), and complementation of the double mutant strain with RNAIII led to a nearly HG003*:ΔrpiRc* phenotype (Fig. S7C). Altogether, these data show that RpiRc is a main player in bacterial growth adaptation upon glucose and fructose supplementation in LB. Also, since these effects are observed only when RNAIII is expressed, it indicates that RNAIII, independently of RpiRc, plays another role in carbohydrate metabolism or other major biochemical pathways involved in *S. aureus* adaptation to glucose-rich conditions.

## DISCUSSION

Although many direct targets of RNAIII are known, our understanding of RNAIII-mediated virulence regulation in *S. aureus* is still incomplete. Here, we identified 53 putative targets of RNAIII using the MS2-TRAP approach (29). Of these, only three known targets of RNAIII (*hla*, *mgrA,* and *rot*) were retrieved in this screen. A reasonable explanation for this is the use of an RNAIII modified by the 5′ addition of an aptamer that can induce a conformational change and by the fact that affinity purification was performed at a single stage of bacterial growth. In this study, we functionally characterized one novel RNAIII target, which is the *rpiRc* RNA. It codes for a regulator of the PPP, a pathway tightly connected with glycolysis (Fig. 8). In its oxidative phase, the PPP transforms glucose-6-phosphate to ribulose-5-phosphate and produces NADPH, making it a major source of reducing equivalents for biosynthetic reactions. In its non-oxidative phase, PPP produces 5-carbon sugars, which are essential for the biosynthesis of nucleic acids and for the synthesis of aromatic amino acids and coenzymes, such as coenzyme A, nicotinamide adenine dinucleotide, and flavine adenine dinucleotide. By acting on the expression level of glucose-6-phosphate dehydrogenase (*zwf* gene) and ribose-5-phosphate isomerase (*rpiA* gene), the TF RpiRc regulates the two paths of the PPP and could favor the virulence of *S. aureus* (21). Several studies show that inactivation of the *rpiRc* gene enhances *S. aureus* virulence in murine infection models (32, 33). The increased virulence appears to be mostly linked to the overexpression of RNAIII found in *rpiRc*-deficient *S. aureus* strains. Indeed, the strong expression of RNAIII efficiently blocks *rot* translation, leading to an increase in toxin production (32).

Usually, RNAIII expression gradually increases to reach a plateau in the late exponential phase of growth (Fig. 1C). Here, it is strongly upregulated in the early-exponential phase of growth when the *rpiRc* gene is inactivated (Fig. S8). Although many data have shown that the pleiotropic TF SarA, a regulator of the Agr quorum-sensing system, is required for the regulation of RNAIII expression mediated by *rpiRc* (21, 33), the mechanism that triggers RNAIII overexpression mainly during the exponential growth phase is unclear. Increased RNAIII expression could result indirectly from the disruption of bacterial metabolism induced by the loss of *rpiRc* expression. Inactivation of *rpiRc* leads to decreased activity of the TCA cycle (32), which could be sensed by specific regulatory proteins, such as CodY and CcpA, which in turn alter the RNAIII expression through the regulation of the Agr quorum-sensing system (23, 28). On the other hand, *S. aureus* expresses three RpiR homologs (RpiRa, RpiRb, and RpiRc). Inactivation of RpiRa in RpiRc-deficient strain restores the expression of *rpiA* and *zwf* but does not alter the *rpiRc*-dependent upregulation of RNAIII (21), showing that partial restoration of PPP does not counteract the overexpression of RNAIII induced by RpiRc deficiency. Given the major role of RNAIII in the expression of *rpiRc* that we show, we can postulate that the absence of the RpiRc protein causes an alarm, which could trigger, through a feedback loop, an increase in RNAIII necessary for the translation of *rpiRc* and activation of the PPP during growth when *S. aureus* needs to increase its DNA, RNA, and protein syntheses.

Indeed, the *rpiRc* mRNA levels are relatively constant during growth [our data and reference (33)] and poorly affected by major virulence regulators in *S. aureus* (33). Additionally, a study focusing on 3′ UTRs revealed that deletion of the *rpiRc* 3′ UTR produces higher RpiRc levels and results in lower levels of hemolysis (34). Since the RpiRc protein contains an N-terminal helix-turn-helix DNA-binding motif and a C-terminal sugar isomerase-sensing domain, it was postulated that RpiRc activity occurs post-translationally and depends on the metabolic status of *S. aureus* (33). Our data show that *rpiRc* activity is not entirely dependent on metabolic stimuli but also requires translational regulation. We demonstrated that the highly structured 5′ UTR of *rpiRc* mRNA prevents the initiation of its translation and that binding of RNAIII to the 5′ UTR of *rpiRc* mRNA allows the translation to occur (Fig. S4). Therefore, RNAIII indirectly increases the expression of PPP enzymes through RpiRc.

Analysis of bacterial growth in LB showed that the deletion of *rpiRc* or *rnaIII* has a major impact on fitness and that only an overexpression RpiRc can compensate for these defects (Fig. 6). Then, we supplemented growth with various carbohydrate substrates involved in PPP or glycolysis (i.e., gluconate, glucose, or fructose, see Fig. 8). All of them significantly modified the growth curves of the different mutants, suggesting that both RpiRc and RNAIII play a role in regulating metabolic fluxes to balance carbohydrate utilization through the PPP and glycolysis. When gluconate was added as the main carbon source, the growth of the HG003*:ΔrpiRc* strain became identical to that of its parental strain in the exponential phase of growth and reached a final biomass closer to the latter (Fig. 7), confirming the role of RpiRc in the regulation of the PPP. When *rnaIII* is inactivated, gluconate does not allow the restoration of a parental exponential phase, indicating that RNAIII regulates other metabolic pathways. Indeed, when growth is carried out in the presence of glucose or fructose, the strains lacking *rnaIII* behave like a parental strain during the exponential phase of growth, which suggest a role for RNAIII in the regulation of glycolysis or TCA. Additionally, glucose and fructose provided an additional positive effect on the final biomass formed by the HG003:*ΔrpiRc* mutant (Fig. 7C and D). Glucose is normally known to induce an *S. aureus* growth defect in the LB medium in a dose-dependent manner (31). This is mainly due to an impairment of the cell wall biosynthesis (31). The positive effect of glucose on the final biomass formed in a strain deleted for *rpiRc* was only observed when RNAIII was expressed (Fig. S7A and C). This could be due to the overexpression of RNAIII in the HG003:*ΔrpiRc* mutant. Indeed, it was reported that RNAIII could affect cell wall integrity by regulating peptidoglycan and LTA biosynthesis (14, 15). However, since we show the disruption of *rpiRc* has an effect on RNAIII expression only during the first hours of growth (Fig. S8), another reasonable explanation would be that RNAIII acts at a broader level, independently of RpiRc. Indeed, PPP was recently shown to be involved in cell wall architecture, leading to a modification of the resistance to β-lactams (35). Since glycolysis, PPP, and cell wall biosynthesis are tightly connected, disruption of RpiRc could alter the cell envelope formation, leading to increased resistance to the negative effect of high concentrations of glucose. *rnaIII* inactivation could lead to further modification of the cell wall structure and thus counteract the positive effect of *rpiRc* inactivation on bacterial growth in the presence of high glucose levels.

Overall, our data show a major role of RpiRc in PPP and suggest that RNAIII is not only an activator of RpiRc but probably regulates other unknown targets that participate in *S. aureus* adaptation to environmental cues. Such a pleiotropic effect of RNAIII on bacterial fitness is not surprising given the multifaceted structure of its RNA sequence and its abundance within the cell. Taken together, we show for the first time that RNAIII can regulate directly and positively one crucial metabolic pathway, the PPP, and influence the metabolic status of *S. aureus*. Since the important role of the PPP in the pathogenicity of S. *aureus* was recently demonstrated (36, 37), regulation of PPP could be another way for RNAIII to influence bacterial virulence. Therefore, this study highlights a new role for RNAIII in the regulatory puzzle that governs *S. aureus* metabolism and pathogenicity.

## MATERIALS AND METHODS

### Bacterial strains, plasmids, and growth conditions

The bacterial strains and plasmids used in this study are listed in Table S2. *Escherichia coli* and *S. aureus* were grown at 37°C and 160 rpm in LB, brain heart infusion (BHI) broth, tryptic soy broth. When indicated, growth mediums were supplemented with glucose, fructose, or gluconate at a concentration of 0.5%. Growth curves were generated from overnight cultures that were diluted to an OD_600nm_ of 0.1, and the optical density of growth was measured using a Bio-Tek 2 (Agilent) instrument. Antibiotics were used at the following concentrations: tetracycline 2 µg/mL; erythromycin 5 µg/mL; chloramphenicol 10 µg/mL; and ampicillin 50 µg/mL. *E. coli* DH5-α was used as the host strain for plasmid construction. Plasmids were propagated in *S. aureus* strain RN4220 prior to transformation in *S. aureus* HG003. All cloning reactions were transformed by heat shock at 42°C into *E. coli* DH5-α and electroporation into *S. aureus* RN4220 (38). All clonings were done with a Gibson Assembly Master Mix (New England Biolabs), which allows a one-step assembly of multiple PCR fragments into a vector. Table S2 lists the primers used and the constructs generated in this study.

For MAPS experiments, two MS2 aptamers were fused to the 5′ end of RNAIII in two-step PCR (Table S2). For the expression of a sole MS2 tag, a *ms2* tag fused to a strong transcriptional terminator sequence was amplified by PCR. PCR products were cloned into pRMC2 (39), an anhydrotetracycline-inducible expression vector, to generate pRMC2-ms2 and pRMC2-ms2-rnaIII plasmids.

The pICS3 vector, a pRMC2 derivative plasmid (13), was used to express *rnaIII* in HG003 strains. For constitutive expression of *RNAIII*, the 41 nt-long P*_amiA_* promoter (40) was cloned upstream of the 5′ end of the genes. For the deletion of the 5′ region of RNAIII, primers were designed to eliminate 60 nucleotides downstream of the seventh nucleotide of RNAIII (pISC3-P*_amiA_-Δ60rnaIII*).

For the GFP fluorescence assay, the pCN33 plasmid (41) was used to construct pCN33-P*tufA-rpiRc-gfp* and pCN33-P*tufA-44rpiRc-gfp* vectors, which express *rpiRc* target under the control of the P*tufA* promoter. The 5′ UTR of *rpiRc* (327 or 44 nucleotides) or the beginning of *rpiRc* coding sequence (first 30 amino acids) were cloned as N-terminal fusions to *gfp*. PCR products corresponding to P*_tufA_* promoter and *rpiRc* or *44rpiRc* were recombined into pCN33-*gfp* plasmid (13) to construct pCN33*-*P*tufA-rpiRc-gfp* and pCN33-P*tufA-44rpiRc-gfp* plasmids.

### Strain construction and gene recombination

Genetically modified strains were obtained by double homologous recombination using the temperature-sensitive vector pIMAY (38). Chromosomal insertion of FLAG epitopes at the C-terminus of RpiRc was performed by generating a *rpiRc-flag* fragment by PCR to obtain pIMAY-*rpiRc-Flag* plasmid.

Deletion of *rpiRc* gene was performed using a Cre-lox-based marker removal system, which enables the removal of the antibiotic resistance marker (TetM) used for the selection of genetically modified *S. aureus* (42). The *tetM* gene encompassed by *lox66* and *lox71* sequences was cloned into the PstI restriction site of the pUC19 plasmid. The *lox66-tetM-lox71* fragment from this plasmid and chromosomal regions upstream and downstream *rnaIII* or *rpiRc* sequences were amplified by PCR and cloned into pIMAY (pIMAY*ΔrnaIII::lox66-tetM-lox71* and pIMAY*ΔrpiRc::lox66-tetM-lox71*). The pIMAY vectors were electroporated into RN4220 and then transferred to HG003. Transformants were initially selected at 28°C for chloramphenicol resistance, encoded by the vector backbone of pIMAY. The subsequent procedure for the isolation of mutants (HG003-*rpiRc-Flag*, HG003*-ΔrnaIII::lox66-tetM-lox71,* and HG003-*ΔrpiRc::lox66-tetM-lox71*) was performed as described (38). Double-crossover events corresponding to the desired gene mutations were confirmed by PCR. Using the method described by Liebig et al. (42), the *tetM* resistance marker used to select mutants was removed by a transient expression of a Cre recombinase (from pRAB1 plasmid), which leads to the replacement of the antibiotic resistance cassette between the two *lox* sites by a DNA sequence containing a 34-bp *lox72* site and 52 nucleotides from pUC19 vector. Inactivation of *rnaIII* gene in HG003-*rpiRc-Flag* and in HG003:*ΔrpiRc* was performed by phage α80 transduction (43) using HG003*-ΔrnaIII::tetM* as donor strain.

### MS2-affinity purification coupled with RNA sequencing

Overnight cultures of HG003 carrying pRMC2-MS2 or pRMC2-MS2-RNAIII vectors were diluted to an OD_600nm_ of 0.1 in 40 mL BHI supplemented with 10 µg/mL chloramphenicol. *S. aureus* was grown to an OD_600nm_ of 5 before the addition of anhydrotetracycline (1 µM final) for 5 min. For crude bacterial extract preparation, cells were pelleted by centrifugation (4,500 rpm, 10 min, 4°C) and resuspended in 2 mL of ice-cold Buffer A (20 mM HEPES pH 7.5, 200 mM NaCl, 1 mM MgCl_2_, and 1 mM DTT). Lysis was carried out with FastPrep apparatus (MP Biomedicals) and 500 µL of 0.1 mm glass beads (Sigma). Lysates were clarified by centrifugation (30,000 × *g*, 10 min, and 4°C). For each sample, 150 µL of amylose resin was precoated with 3 nmoles of MS2-maltose-binding protein purified as described (44) in buffer A and incubated for 10 min at 4°C with 1.5 mL of clarified lysates in 2 mL Eppendorf tube. Beads were washed seven times by centrifugation (13,000 × *g*, 2 min) with 1 mL of Buffer A. RNAs were eluted with 300 μL of buffer A containing 50 mM maltose. Eluted RNAs were extracted with phenol/chloroform:isoamylalcohol (25:24:1) and precipitated with three volumes of cold absolute ethanol in the presence of 0.3 M sodium acetate. The RNA samples were treated with Amplification Grade DNase I (Thermofisher, USA) prior to RNA-seq. The efficiency of the DNA treatment was verified by qPCR. RNA was quantified using Qubit (Life Technologies), and the integrity was assessed with a Bioanalyzer (Agilent Technologies). Stranded cDNA libraries were generated using the NEBNext Ultra Directional RNA Library Prep Kit for Illumina (New England Biolabs, USA) following the manufacturer’s recommendations. The concentration, quality, and purity of the libraries were determined using BioAnalyzer, a Qubit fluorometer (Invitrogen, USA), and a Nanodrop spectrophotometer (Thermo Scientific, USA). Libraries were sequenced on an Illumina MiSeq instrument (paired end, 150 cycles) as per the manufacturer’s instructions. Quality control of RNA-Seq reads and read mapping onto *S. aureus* NCTC8325 genome were performed as previously described (45). SAM files were filtered on bitwise flag values (46), and properly paired fragments were counted by HTSeq with the intersection nonempty mode (47) and using an NCTC8325 annotation file (in GFF format) obtained from NCBI. Enrichment of mRNA targets was calculated using DESeq under the per-condition mode (48).

### Fluorescence reporter assay

HG003*ΔrnaIII* carrying pCN33-target-RNA-GFP fusions and RNAIII variants, cloned into the pICS3 vector, were grown overnight at 37°C in BHI supplemented with 10 µg/mL chloramphenicol and 5 µg/mL erythromycin. Strains were streaked on a BHI-antibiotics agar plate and grown overnight at 37°C to measure fluorescence by scanning bacteria at 473 nm with a Typhoon device (GE Healthcare) using LPG filters. Growth (control) was monitored by scanning the colonies with LPG filters at 532 nm in visible light. For the analysis of GFP expression in liquid culture, overnight cultures were diluted to an OD_600nm_ of 0.1 in BHI medium containing both antibiotics. Triplicates of 150 µL cultures were placed in a 96-well microtiter plate and incubated in a Synergy 2 Multi-Mode Reader (BioTek) at 37°C under continuous shaking. Bacterial growth and GFP expression were monitored every 10 min for 20 h by measuring absorbance at 600 nm and fluorescence using a 485/20 nm excitation filter and a 528/20 nm emission filter (tungsten lamp). The average fluorescence and standard deviation were calculated from three independent experiments.

### *In vitro* transcription, gel retardation, and toeprint assays

DNA template containing T7 promoter sequence upstream *rnaIII*, *rpiRc,* and their variants was generated by PCR using primers listed in Table S2. PCR products were used as templates for *in vitro* transcription performed with a MEGAscript T7 kit (Ambion). RNAs were separated on an 8% polyacrylamide-7 M urea gel electrophoresis and eluted overnight in G50 elution buffer (20 mM Tris-HCl pH 7.5, 2 mM EDTA, and 0.25% SDS). RNAs were precipitated in cold ethanol and 0.3 M of sodium acetate and then dephosphorylated using Calf-intestinal alkaline phosphatase (New England Biolabs) according to the manufacturer’s protocol. The 5′ end labeling of RNA was obtained with T4 polynucleotide kinase (New England Biolabs) and [γ^32^P] adenosine triphosphate as previously described (49). Gel retardation and toeprint assays were performed as previously described (50). Briefly, electrophoretic mobility shift assays were performed with either the native RNAs or the RNAs deleted for their predicted interaction domains. Labeled *rpiRc* mRNA or RNAIII of 0.3 nM concentration was incubated with various concentrations (from 75 to 2,400 nM) of unlabeled RNAs and loaded on a 6% polyacrylamide gel under non-denaturing conditions. Gels were dried and visualized using a Typhoon Phosphorimager (Molecular Dynamic), and the data were quantified with ImageQuant software (GE Healthcare Life Science). The dissociation constant (kd) was evaluated as the concentration of the cold RNA leading to 50% of binding. cDNAs generated during the toeprint assay were separated in 8% denaturing polyacrylamide gel electrophoresis. Gels were dried and visualized using a Typhoon Phosphorimager (Molecular Dynamic)

### Bacterial RNA isolation, RNA half-life determinations, and relative expression of genes by RT-qPCR

For total bacterial RNA isolation, cell suspensions were centrifuged at 13,000 × *g* for 1 min at 4°C. The pellet was resuspended in 500 µL lysis buffer (0.5% SDS, 20 mM sodium acetate, and 1 mM EDTA, pH 5.5) and transferred into a FastPrep bead beater tube containing 500 µL of 0.1 mm glass beads and 500 µL of phenol (pH 4). Cells were broken for 30 s at a power of 6.5 in a FastPrep-24 5G instrument (MP Biomedicals), and cellular debris was removed by centrifugation. Total RNA was isolated from the supernatant by phenol-chloroform extraction and ethanol precipitation as described (51).

For RNA half-life determination, *S. aureus* was cultured overnight, diluted to an OD_600nm_ of 0.1, and grown for 5 h in BHI medium. The transcription was stopped by the addition of 200 µg/mL (final) of rifampicin. Six hundred microliters was removed before rifampicin treatment (time zero) and at different incubation times after treatment. Then, the samples were combined with an equal volume of cold acetone:ethanol (1:1, vol/vol, −20°C) and centrifuged. RNAs were extracted from the pellet as described above and quantified using a NanoDrop 2000 spectrophotometer (ThermoFisher Scientific). For quantitative real-time PCR assay, 2 µg of RNA was treated with 3 units of DNase I amplification grade (Invitrogen, Carlsbad, USA) for 20 min at 22°C to remove any DNA contaminants. The High-Capacity cDNA Archive Kit (Applied Biosystems, Foster City, USA) was used to convert RNA into cDNA, which was amplified using a Power SYBR Green PCR Master mix (Applied Biosystems) (5′ PRIME, Life Technologies, Carlsbad, USA) on a StepOnePlus Real-Time PCR system (GE Healthcare, Saint Aubin, France) with the primers listed in Table S2. Relative transcript levels were calculated with the ΔΔCt method using *gyrB* mRNA, *16S* rRNA, or *tmRNA* as internal controls. The half-life of each transcript was calculated as the time point at which the RNA level was decreased by a factor of 2 as compared to the RNA level measured before rifampicin treatment (time zero).

Northern blot was done using 10 µg total RNA as previously described (52). The membrane was hybridized with specific ^32^P-labeled probes (sequences listed in Table S2) in ExpressHyb solution (Clontech) for 60 min at 37°C. The membrane was washed, exposed, and scanned with a Typhoon FLA 9500 phosphorimager.

### Protein purification and Western blots

For total protein extracts, cell pellets corresponding to 5 units of OD_600nm_ from *S. aureus* were resuspended into 150 µL of lysis buffer L (10 mM HEPES pH 7.5, 150 mM NaCl, 2 mM MgCl_2_, and 1 mM EDTA) containing 0.1 mg/mL lysostaphin and EDTA-free protease inhibitor cocktail (Roche). Following incubation at 37°C for 10 min, 20 µL of 10% SDS was added. Samples were boiled for 5 min and centrifuged for 5 min at 13,000 × *g*. Protein quantity in the supernatant was measured using the Qubit protein assay kit (Invitrogen). Laemmli sample buffer was added, and 50 µg of proteins was separated by SDS-PAGE and transferred onto a hybond-P polyvinylidene fluoride membrane (Amersham). RpiRc-FLAG protein was detected with horseradish peroxidase-conjugated anti-FLAG antibodies (Sigma-Aldrich). Western blot was revealed using the Amersham ECL Plus Detection Kit and visualized using LAS 4000 (GE Healthcare).

## Data Availability

RNAseq reads were submitted to SRA under the following BioProject accession number: PRJNA803435.

## SUPPLEMENTAL MATERIAL

Supplemental figures msphere.00348-23-s0001.pdf Figures S1 to S8.

Table S1 msphere.00348-23-s0002.xlsx mRNAs enriched by affinity purification.

Table S2 msphere.00348-23-s0003.xlsx Strains, plasmids, and primers used in this study.

ASM does not own the copyrights to Supplemental Material that may be linked to, or accessed through, an article. The authors have granted ASM a non-exclusive, world-wide license to publish the Supplemental Material files. Please contact the corresponding author directly for reuse.
