# Supplementary material for: RNAIII is linked with the pentose phosphate pathway through the activation of RpiRc in Staphylococcus aureus
Source: mSphere. 2024 Apr 9;9(5):e00348-23. doi: 10.1128/msphere.00348-23 (PMC11237564; doi:10.1128/msphere.00348-23)
Supplement: Supplemental figures — Figures S1 to S8. [file msphere.00348-23-s0001.pdf]

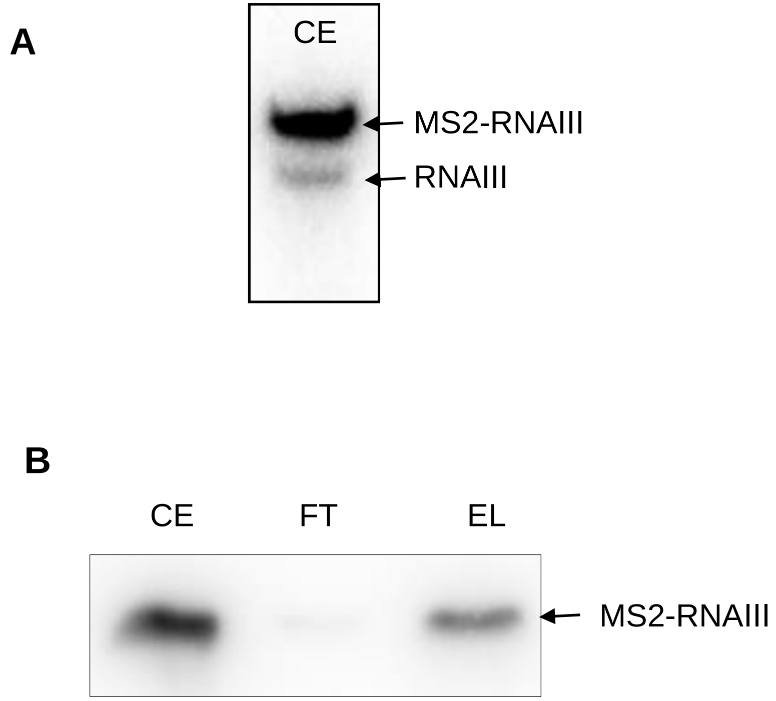

Figure S1: The MS2-RNAIII variant is over-expressed and retained by affinity chromatography. (A) Northern blot showing the expression of RNAIII and MS2-RNAIII in HG003 strain using 5'-labelled RNAIII probe. (B) Northern blot targeting MS2-RNAIII with 5'-labelled MS2 probe during purification on MS2 chromatography affinity. RNAs were extracted from a same volume of crude extract (CE), flow-through (FT) and elution (EL), and loaded on urea polyacrylamide gel.

```
     -320                -300                -280                -260
5’AAUGAUUAAGAUGGUAGAUUUAGUCUUAUAUAACUACAUUUACUAAGAUUUAAAUAGAUGAGUACAUA

               -240                -220                -200
AUGUAUAAAUUGGGAAUAUACAGUAUGACUUACGGUAAUAUAUCAAUAUAUUGAUAUAUUAUUUGCACAU

      -180                -160                -140                -120
GGGAGUAUGCUAUUUUUUAUCUAUACAUCUUAAAAAAAAUUUACAGUUUUACAAUUCAAUAUCAUUAAACA
                          -161
               -100                -80                 -60
AACAACAUAUUCAGCUUAUUUUUCAUUCUCUUACGUUACAAGUUGUAAAUACCAUCGCGUUAAACAACAA

      -40                 -20                 +1
UAGCCAACGCUAAUAAAAUAUAUUGCGUUAAAGUGGAGUGAUUAAUAUGUCAAACGUACUAACAGAAAUA
  -44                            SD           Start

GAUAGUCAAUAUCCAUAUAUGACUAAAAAUGAAAAAAAGAUAGCUAAAUUUAUACUAAAUUCACCUCAAA

AGGUGAUUAAAAUGAGGUCUCAAGACUUAGCAAGCUUAUUAGAUAUUAGUACAUCAUCAGUCAUUCGAUU

CAGCAAGAAAAUUACUGAUGGCGGUUUUCACGACUUGAAAAUAAAUAUAUCUAAAUAUGUUCCUAAAGCA

UCAUCAAUUUACAAUGUUGAAUUAAUGAAUAACGAAAGCACAGAAUCUUUAAGAACUAAACUCCAUACAC

GUACUACACGCGCACUUAAUCAUGCAAAUAACGAAUUAAAUGAUAAAACUAUUGAUCAAAUAUGUCAUUG

UUUAAAACGUUCUGAGACGAUUUUUAUAUAUGGUUUUGGCGCAUCUUUUGUAGUUGCUACCGACUUAUAC

CAAAAGUUAUCAAGAAUAGGUCUUAAUAUUCAACUUGUUCAAGAAACACAUAUUUUUGCUACAUUAUUAG

CAACUCACAAUUCGAACGACAGUGUUAUUCUUAUUACUAACAAUGGUACGCAAAGUGAAAUGCAAUCAAU

GGUUAAAGUUAUUGAUGACUACCAUAUACCUAUAAUUACAAUUACUAGUACAAGGGAUAAUCCCGUGCAC

AGGCAUCAAAUAUUGUUUUAACUUAUGGGAAAACUGAUGAAAAUGAGAUGCAUAUGGGAGCAACAACUUC

AUUAUUUGCUCAAAUGUUCACUAUUGAUAUUUUAUAUUAUCGUUAUGUUGCAUUAAAUUAUCACGCAUCA

UUAGAUUUCAUAACACAAUCUAAAAUGGCUUUAGAUAACUAUCGUAAACAUUUAUCAAAUAUUAACUUUA

AACAUUAACAGUCUUAUAUAAAUUCUUUAAUCAUGUCAUAUAAUAAUUUAUGCAUCAUUUUCAAAGCUUA
     Stop
CAUGUCUUUUAUCUUGUAUAACAUAC 3’
```

Figure S2: Nucleotide sequence of *rpiRc* mRNA. The 5’and 3’ end of *rpiRc* mRNA were determined by RACE. The Shine-Dalgarno sequence (SD), the AUG start codon and UAA stop codon are underlined. Region of *rpiRc i*nteracting with RNAIII is indicated in green.

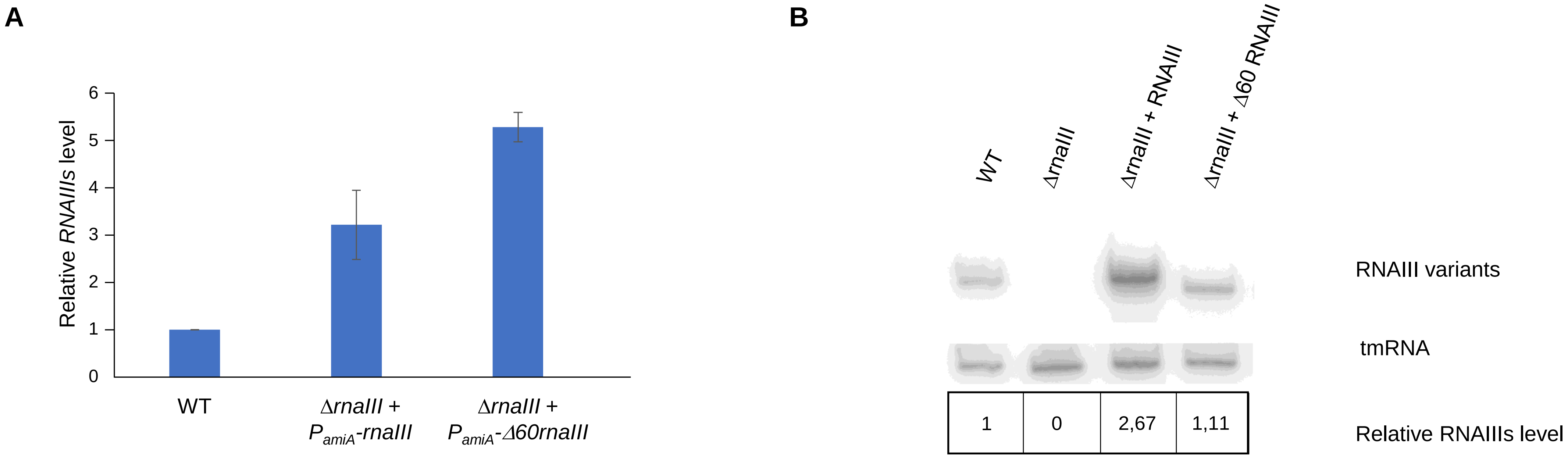

Figure S3: Expression levels of RNAIII variants transcribed from plasmids under the control of a constitutive or endogenous promoter. (**A**) Expression levels of RNAIII variants expressed from pISC3 plasmid under the control of the P*amiA* constitutive promoter. The RNAIII variants were quantified by RT-qPCR. The data were normalized to the level of *gyrB* mRNA expression from total extracts prepared from an early-stationary phase culture (6h) of HG003 (WT) and *Δrnalll* strains complemented with a plasmid expressing either RNAIII or Δ60RNAIII under the constitutive P*amiA* promoter. The data obtained from WT strain was set to 1 for comparison. (**B**) Expression levels of RNAIII variants expressed from pISC3 plasmid under the control of the endogenous RNAIII promoter. Total RNA from HG003 (WT) and *Δrnalll* strains complemented with a plasmid expressing either RNAIII or Δ60RNAIII under the endogenous RNAIII promoter was isolated at the early-stationary phase of growth (6h). RNAs were separated on 8% urea-PAGE, transferred to a positively charged membrane and probed with radiolabelled oligonucleotides specifics to RNAIII or *tmRNA*. The Northern blots were scanned using a Typhoon FLA 9500 phosphoimager and quantified with ImageJ software. The data were normalized to *tmRNA* expression level. The normalized expression was set to 1 for WT strain.

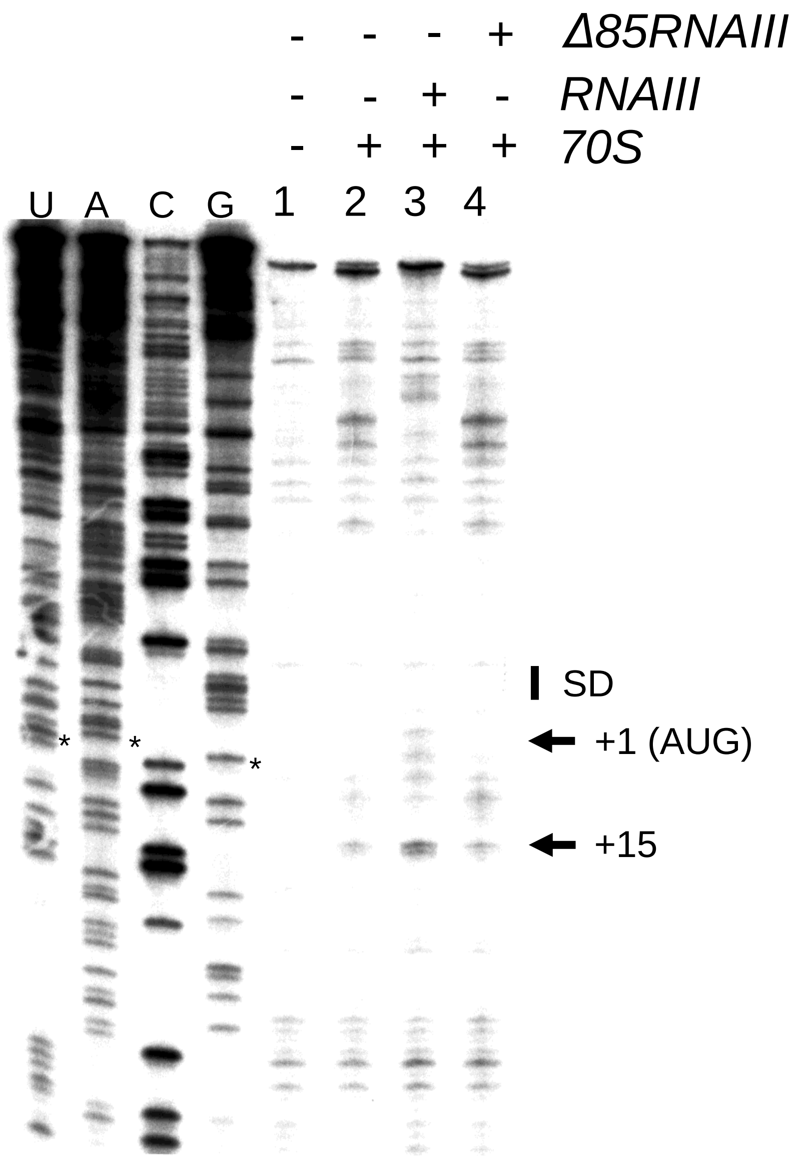

Figure S4: *In vitro* Toeprint assay to monitor the effect of RNAIII on the initiation of ribosomal complex formation. The *rpiRc* mRNA was incubated with *S. aureus* 70S, the initiator tRNAfMet and either RNAIII or *Δ*85RNAIII, an RNAIII deprived of its *rpiRc*-interacting domain. The experiment shows that the addition of RNAIII favors the formation of the initiation complex to the AUG site of *rpiRc* mRNA. The Shine-Dalgarno sequence, the start site of translation (AUG *) and the toeprint signals +1 and +15 of the AUG codon are indicated. C, G, U, A: sequencing ladders.

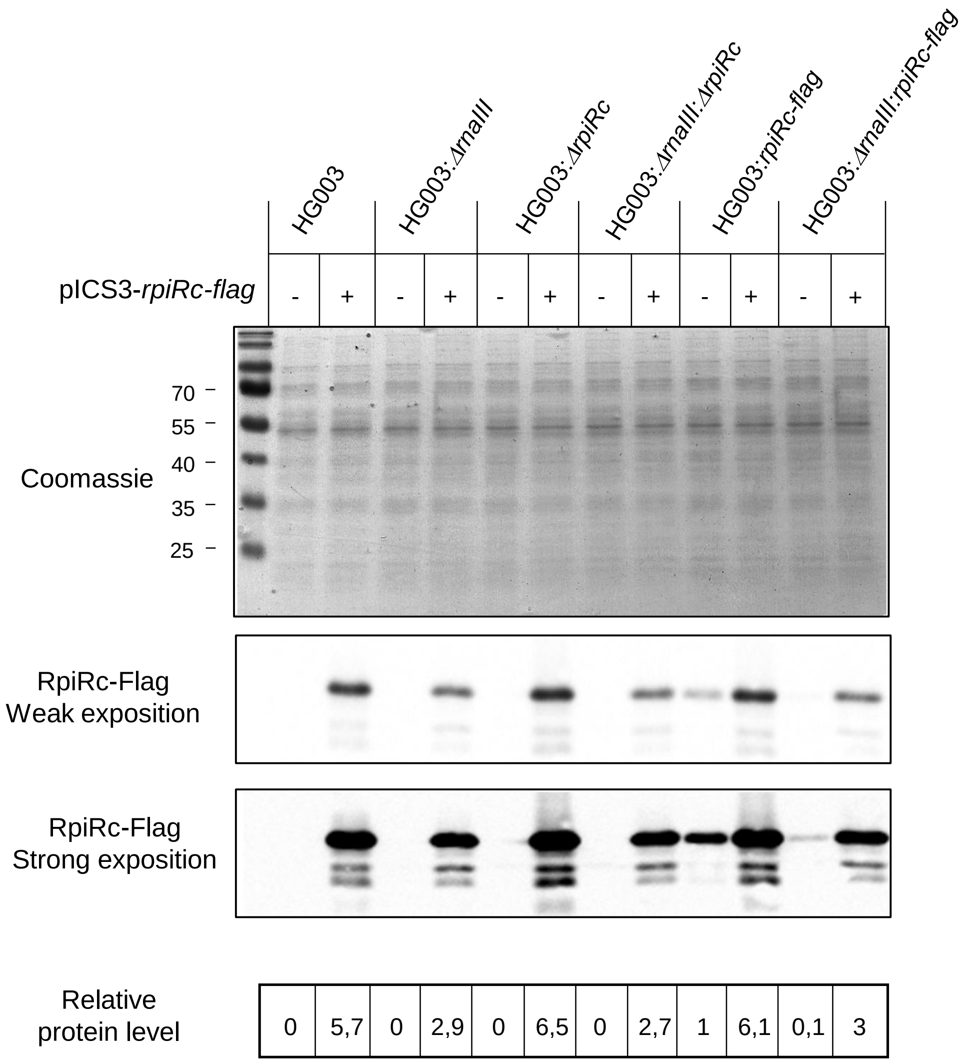

Figure S5: Complementation with *rpiRc* leads to an over-expression of RpiRc in HG003 mutants. To monitor the expression of RpiRc under its own endogenous promoter in the pICS3 vector, the RpiRc protein was fused to a Flag epitope at its C-terminus. The expression level of FLAG-tagged RpiRc proteins in HG003, HG003:Δ*rnaIII*, HG003:Δ*rpiRc*, HG003:Δ*rnaIII*:Δ*rpiRc*, HG003:*rpiRc-flag* and HG003:Δ*rnaIII*:*rpiRc-flag* strains containing pICS3 or pICS3-*rpiRc-flag* vectors was determined after 4 h of growth in TSB. FLAG-tagged RpiRc proteins were detected by immunoblotting with anti-FLAG antibodies. The relative expression of RpiRc-FLAG was normalized to 1 in HG003:*rpiRc-flag* strain HG003 (HG003:*rpiRc-flag* with pICS3). Coomassie blue stained SDS-page gel is included to demonstrate the equivalent loading of total proteins.

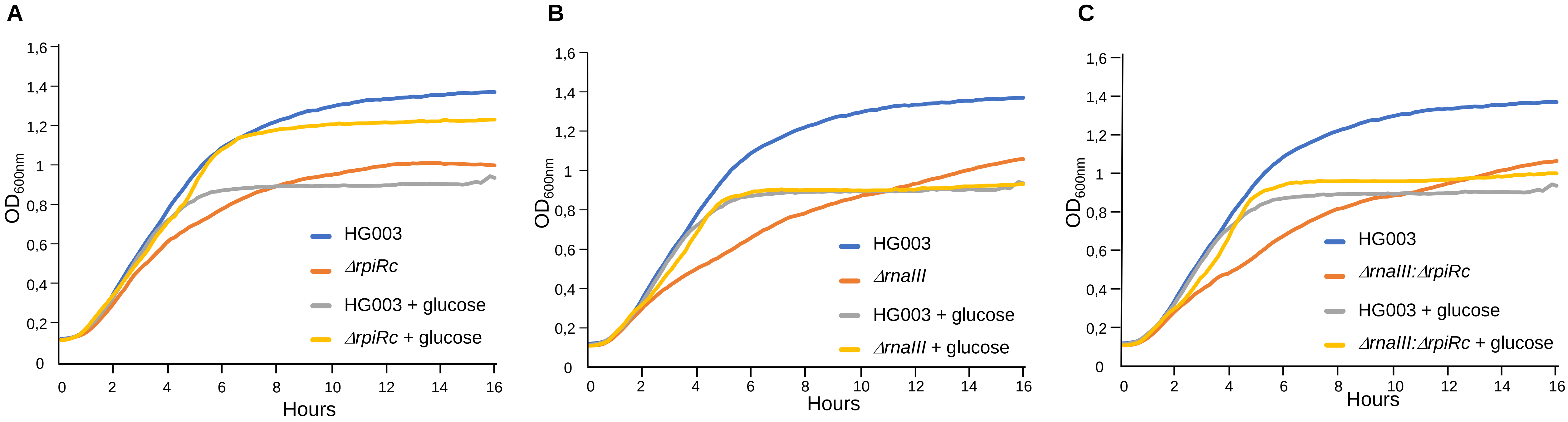

Figure S6: Effect of glucose on the growth of **(A)** HG003:Δ*rpiRc*, **(B)** HG003: Δ*rnaIII* and **(C)** HG003: Δ*rnaIII:* Δ*rpiRc* in LB medium. In each panel, the growth was compared with the parental strain HG003.

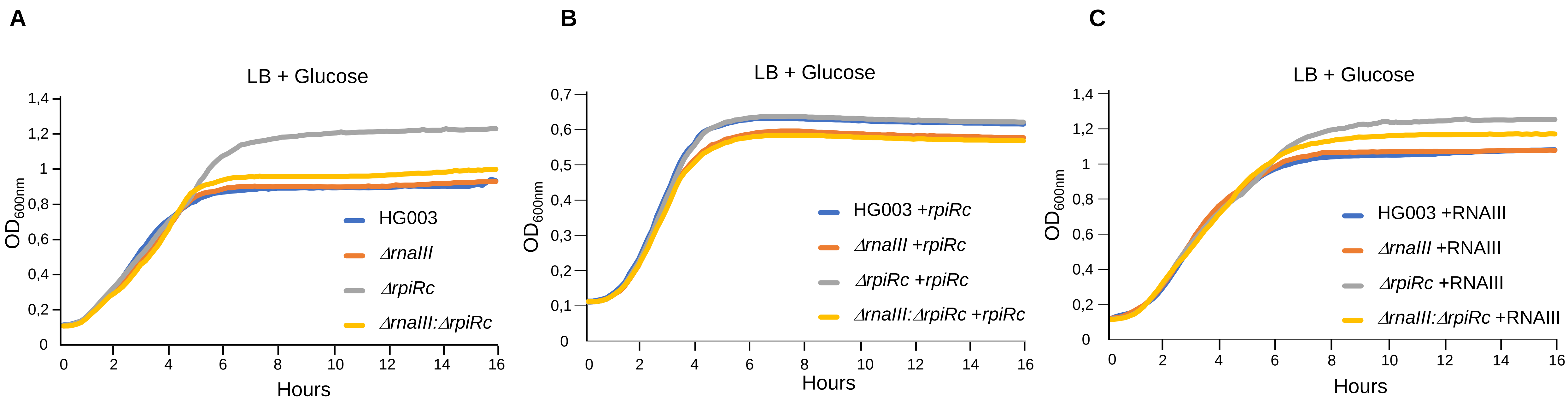

Figure S7: Growth of HG003 and its *rnaIII* and/or *rpiRc* mutants complemented with **(A)** pICS3 **(B)** pICS3-*rpiRc* (+*rpiRc*) or **(C)** pICS3-*tufA-rnaIII* (+RNAIII) in LB medium supplemented with 0,5% of glucose.

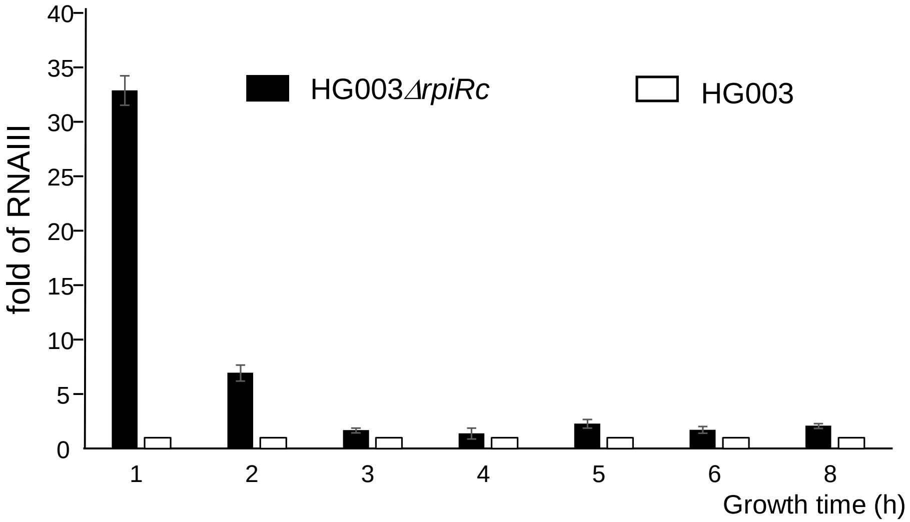

Figure S8: Relative expression of RNAIII in HG003*:ΔrpiRc*. Inactivation of *rpiRc* gene leads to an upregulation of the expression of RNAIII in the early-exponential phase of bacterial growth. The levels of RNAIII were quantified by RT-qPCR. The data were normalized to the level of *gyrB* mRNA. The error bars show standard errors of the means of three independent experiments**.**
